# Defect Visualization of a Steel Structure Using a Piezoelectric Line Sensor Based on Laser Ultrasonic Guided Wave

**DOI:** 10.3390/ma12233992

**Published:** 2019-12-02

**Authors:** Sang-Hyeon Kang, Dae-Hyun Han, Lae-Hyong Kang

**Affiliations:** 1 Department of Mechatronics Engineering, Jeonbuk National University, 567 Baekje-daero, Deokjin-gu, Jeonju-si 54896, Korea; shkang82@jbnu.ac.kr (S.-H.K.); dh.han@jbnu.ac.kr (D.-H.H.); 2 LANL-JBNU Engineering Institute-Korea, Jeonbuk National University, 567 Baekje-daero, Deokjin-gu, Jeonju-si 54896, Korea; 3 Department of Flexible and Printable Electronics, Jeonbuk National University, 567 Baekje-daero, Deokjin-gu, Jeonju-si 54896, Korea

**Keywords:** defect visualization, piezoelectric, line sensor, laser ultrasonics, guided wave

## Abstract

We studied the detection and visualization of defects in a test object using a laser ultrasonic guided wave. The scan area is irradiated by a laser generated from a Nd:YAG 532 nm Q-switched laser generator through a galvanometer scanner. The laser irradiation causes the surface temperature to suddenly rise and then become temporarily adiabatic. The locally heated region reaches thermal equilibrium with the surroundings. In other words, heat energy propagates inside the object in the form of elastic energy through adiabatic expansion. This thermoelastic wave is typically acquired by a piezoelectric sensor, which is sensitive in the ultrasonic domain. A single piezoelectric sensor has limited coverage in the scan area, while multi-channel piezoelectric sensors require many sensors, large-scale wiring, and many channeling devices for use and installation. In addition, the sensors may not acquire signals due to their installed locations, and the efficiency may be reduced because of the overlap between the sensing areas of multiple sensors. For these reasons, the concept of a piezoelectric line sensor is adopted in this study for the first time. To verify the feasibility of the line sensor, I- and L-shaped sensors were attached to a steel structure, and the ultrasound signal from laser excitation was obtained. If the steel structure has defects on the back, the ultrasonic propagation image will be distorted in the defect area. Thus, we can detect the defects easily from the visualization image. Three defects were simulated for the test. The results show that the piezoelectric line sensor can detect defects more precisely and accurately compared to a single piezoelectric sensor.

## 1. Introduction

Non-destructive testing is carried out in various industries such as aviation, transportation, shipbuilding, and power generation to constantly ensure the safety and maintain the performance of aircraft, machinery, equipment, and structures. The non-destructive testing (NDT) and inspection market is expected to grow from USD 8.3 billion in 2018 to USD 12.6 billion by 2024 at a compound annual growth rate (CAGR) of 7.24% from 2018 to 2024 [1]. Stringent government regulations regarding public safety and product quality, and continuous advances in electronics, automation, and robotics are among a few major factors driving the growth of the NDT and inspection market. The growth of the NDT and inspection market is also propelled by the high adoption rate of Internet of Things (IoT) devices, and the increasing need to assess the health of aging assets [1]. Traditional inspections of material are mainly made by cutting or visual inspection, but the cut material loses its product value, whereas visual inspection does not reveal internal defects. In contrast, non-destructive testing has the advantage of being able to inspect the internal conditions without altering the original form or function of the material or product. Non-destructive testing refers to all forms of inspection that identify the existence, condition, and character of defects without destroying, separating or damaging the specimens through special methods exploiting physical phenomena in the materials or products. Non-destructive testing capable of detecting defects is hence indispensable. In addition, it is important to ascertain the safety of aircraft in order to prevent accidents from severe conditions such as extreme temperatures, high pressure, and high speed. Many non-contact non-destructive testing systems are being developed for the large-scale inspection of large-sized aircraft structures to ensure worker safety [2,3,4,5].

Non-destructive testing methods include visual testing (VT) [3], penetration testing (PT) [3], magnetic particle testing (MT) [3], radiation testing (RT) [3], eddy current testing (ET) [3,6], leak testing (LT) [3], and ultrasonic testing (UT) [3,7]. Each technique has its own advantages and disadvantages [3]. VT, PT, and MT can only detect surface cracks. Radiation testing is harmful to humans. ET can only be used if the specimen is a conductor. LT is suitable for the inspection of discontinuities in the specimen. UT requires liquid couplant to efficiently transmit ultrasonic signals to the specimen. Non-destructive testing techniques have been developed based on desirable characteristics such as real-time inspection, low cost, high efficiency, and high precision. Other non-destructive testing methods such as phased array ultrasonic testing and time of flight diffraction ultrasonic testing are mainly used to inspect welded pipes in nuclear power plants [8]. The infrared thermographic method has the advantages of wide range and fast non-contact inspection [9]. Care should be taken in active thermography to avoid damage to the tested object from excessive thermal loading [10]. Terahertz imaging technology has high resolution and is harmless. However, the nature of electromagnetic waves in terahertz imaging makes it difficult to inspect conductive materials [4,5]. Laser ultrasonic testing has advantages over UT in that it can be performed in real-time and is non-contact, and does not require a liquid couplant [11,12]. Further, the long inspection distance and the freedom to position the excitation source and sensor arbitrarily render laser ultrasonic testing particularly suitable for our final goal of inspecting large structures [11,12].

**Figure 1:**
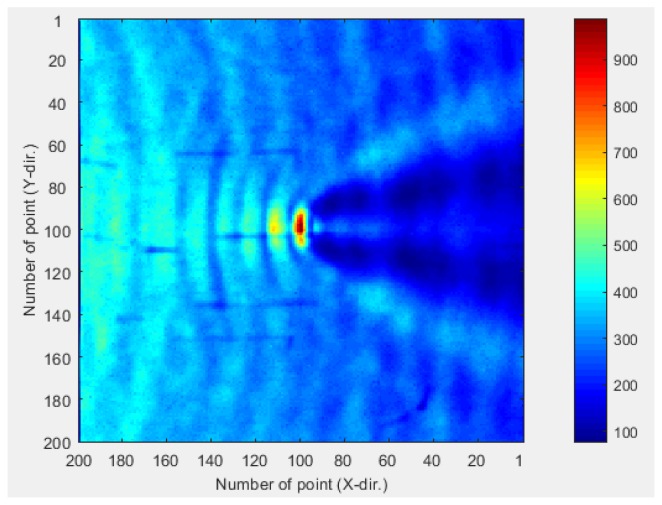
Power spectral density distorted by the defect showing the back of the defect [14].

**Figure 2:**
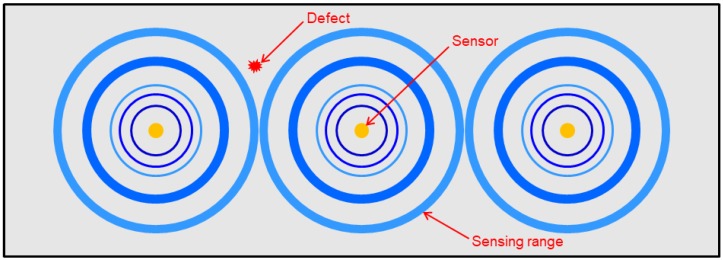
Schematic illustration of non-detection of a defect falling outside the sensing areas of multiple sensors.

A single piezoelectric sensor has limited coverage in the sensing area. Multi-channel piezoelectric sensors require multiple sensors, large-scale wiring, and many channeling devices for installation [13]. In addition, the signal may be distorted by defects, and the positioning of sensors when multiple sensors are installed may result in regions where ultrasonic waves cannot be acquired [14]. The sensor areas may also overlap, reducing the sensor efficiency. This limitation can be overcome by using a single piezoelectric line sensor. Figure 1 shows the power spectral density distorted by the defect in a previous study, and Figure 2 shows that there are areas in which defects are difficult to detect depending on the positions of the sensors.

In this study, a piezoelectric line sensor using PZT5A3 was adopted to overcome the limitations of single piezoelectric sensors and multi-channel piezoelectric sensors, and an algorithm was applied to improve the accuracy and visualization of defect detection [4,5]. The inspected object was irradiated by a laser generated by a laser ultrasonic testing system. The laser ultrasonic guided wave generated at the object was acquired by the piezoelectric line sensor, and the visualization algorithm was applied to detect and visualize defects.

## 2. Principle of Laser Ultrasonic Generation

### 2.1. Mechanism of Laser Ultrasonics

**Figure 3:**
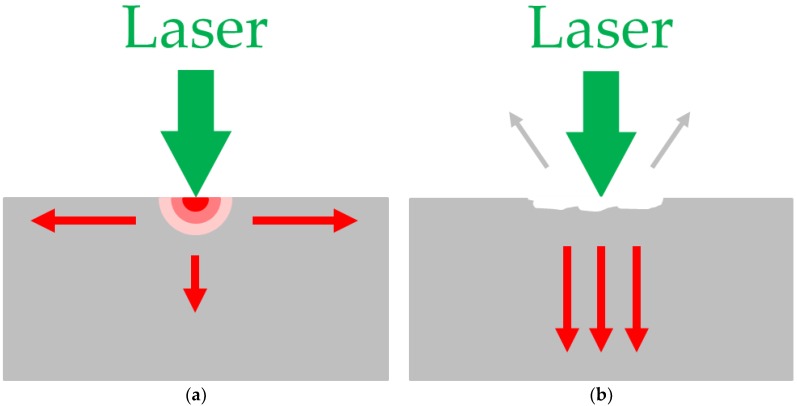
Mechanism of laser ultrasonics: (**a**) thermoelastic regime and (**b**) ablation regime.

Energy absorption and reflection occur in a very thin absorbing layer of a solid surface when a high power laser pulse irradiates the surface. The absorbed energy results in a temperature gradient in which the temperature of the surface rises and falls within a very short time. Due to the creation of the temperature gradient in a very short time, the thermoelastic effect causes an instantaneous expansion of the material. High-frequency thermoelastic stress and strain are hence transferred into the solid specimen. Various physical phenomena appear when the laser irradiates the solid surface. If the intensity of the incident light is low, the temperature of the test specimen rises, causing thermal and elastic waves. If the intensity of the incident light is high, melting, plastic deformation, and cracks occur inside the test specimen, and ablation causes the formation of plasma on the surface. Figure 3 shows the generation mechanism of laser ultrasonics [15,16,17,18].

### 2.2. Thermoelastic Regime

When a low-power laser irradiates a solid surface, absorption of the laser occurs at the surface. Since the absorbed laser consists of electromagnetic waves, the free electrons on the solid surface are vibrated by the absorbed laser, causing a local increase in the temperature of the solid surface. The change of the surface temperature at this time depends on the duration of the laser pulse. If the laser pulse width is long enough, the thermal energy on the solid surface diffuses into the solid, causing the surface to cool to its original temperature or to generate heat waves. Alternatively, if the laser pulse width is very short, the surface temperature rises rapidly and then becomes an adiabatic state. The locally heated portion thus reaches thermal equilibrium with the surroundings through adiabatic expansion. Thermal energy is therefore propagated inside the specimen in the form of elastic energy through adiabatic expansion. This occurs when the incident power of the laser falls below the threshold for the permanent deformation of the material, and no defects or traces are left on the surface. This state is called the thermoelastic regime [15,16,17,18].

### 2.3. Ablation Regime

As the laser power increases, ultrasonic waves are generated by the reaction force resulting from the ablation of the specimen surface when the specimen surface temperature reaches the vaporization point. In this case, unlike the thermoelastic regime, the high incident power causes the vaporization of the material on the specimen surface or the radiation of ions and electrons from the solid specimen to form a blue flame in the visible region. Melting, plastic deformation, and cracks may result in the specimen. Such a regime is called the ablation regime. In contrast to the incidence of low laser power that does not cause damage, the incidence of high laser power involves damage to the specimen surface [15,16,17,18,19].

## 3. Laser Ultrasonic Guided Wave

A guided wave is defined in this section as an ultrasonic wave where the section of an isotropic homogeneous plate with free surface boundary condition is parallel to the wave propagation direction under plane strain condition of two-dimensional elasticity. Guided waves are used in non-destructive testing to detect defects in extended structures. They can travel for long distances with little energy loss. Nowadays, guided waves are widely used to inspect and screen many engineering structures, in particular metallic pipelines around the world. In some cases, hundreds of meters can be inspected from a single location. There are also some applications for the inspection of rail tracks, rods and metal plate structures [2,15,16].

Laser ultrasonic guided waves occur in the thermoelastic regime, do not damage the specimen surface, and are useful for detecting defects in plates and tubes. The laser ultrasonic guided wave method is a non-destructive testing method which has almost no restrictions on the medium to be inspected and can be adjusted to a very wide frequency band. It has the advantage that the non-contact distance is much longer than conventional methods [17,18].

## 4. Defects Visualization Using I-Shaped Sensor

### 4.1. Experimental Setup Using I-Shaped Sensor

**Figure 4:**
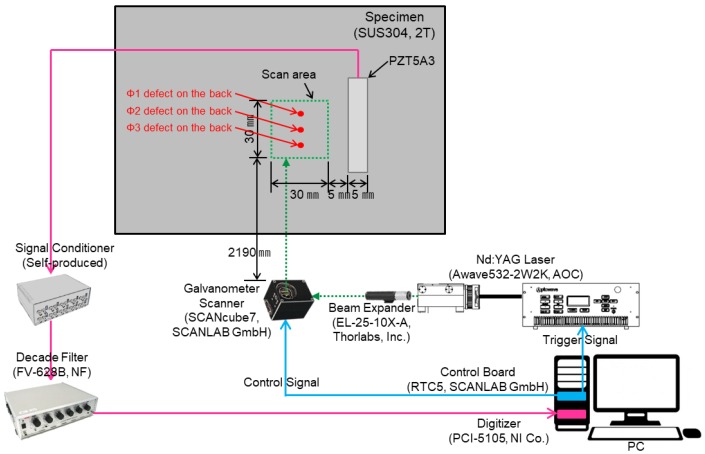
Schematic of the experimental setup using the I-shaped sensor.

**Figure 5:**
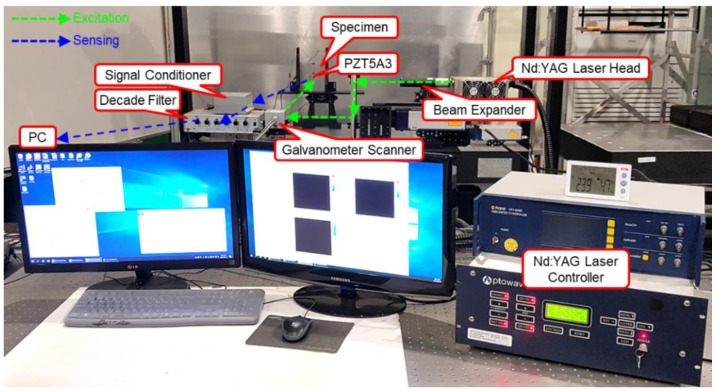
Photograph of the experimental setup using the I-shaped sensor.

Defect visualization using laser ultrasonic waves is performed by acquiring the ultrasonic waves generated from the surface of a structure and imaging the acquired signals to visualize defects. In order to detect defects, a Nd:YAG 532 nm Q-switched DPSS (diode-pumped solid-state) laser generator and a PZT5A3 sensor were used. The thickness of the PZT5A3 sensor and stainless steel specimen are 0.25 mm and 2 mm respectively. The defects of ϕ1, ϕ2, and ϕ3 sizes were made on the back of the specimen without penetrating the specimen, and the I-shaped PZT5A3 sensor was attached to the specimen. The pulsed laser generated by a laser generator with a beam diameter of 0.5 mm was scanned along the x and y coordinates on the scan area at 0.5 mm intervals using a galvanometer scanner, and can detect a defect larger than 1 mm. The thermoelastic wave generated by thermal expansion in the specimen was detected with the PZT5A3 sensor. The signal acquired from the PZT5A3 sensor was amplified by a self-produced signal conditioner, and the noise was removed through a filter. The signal with reduced noise is acquired by a digitizer and visualized by applying a signal processing algorithm. Figure 4 and Figure 5 show the schematic and the photograph of the experimental setup respectively.

### 4.2. Experimental Results Using the I-Shaped Sensor

**Figure 6:**
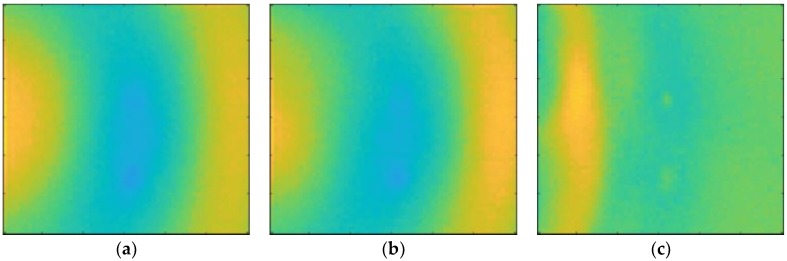
Ultrasonic propagation images at different band-pass filter bandwidths: (**a**) 5 kHz–50 kHz; (**b**) 5 kHz–100 kHz; and (**c**) 5 kHz–500 kHz.

**Figure 7:**
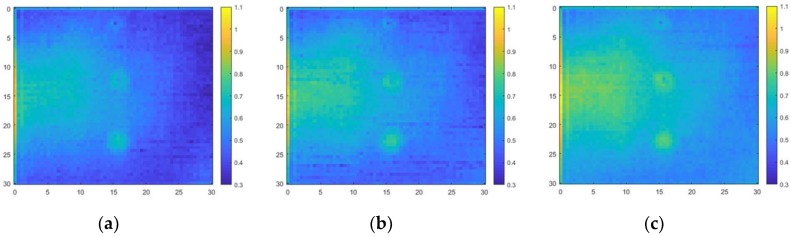
Power spectral density at different band-pass filter bandwidths: (**a**) 5 kHz–50 kHz; (**b**) 5 kHz–100 kHz; and (**c**) 5 kHz–500 kHz.

The ultrasonic waves acquired by the sensor through the signal conditioner and filter were visualized using ultrasonic propagation images and the power spectral density. Ultrasonic propagation images were shown in the time-domain and the power spectral density was obtained using Parseval’s theorem. The results are shown in Figure 6 and Figure 7. These are shown at different bandwidths of the band-pass filter (BPF). It is difficult for non-professionals to locate defects from ultrasonic propagation images. The defects are more easily distinguished through the power spectral density than from ultrasonic propagation images. The three defects were hence detected. The defects may also be difficult to identify if they are obscured by shadows behind the defects.

## 5. Defects Visualization Using the L-Shaped Sensor

### 5.1. Experimental Setup Using the L-Shaped Sensor

**Figure 8:**
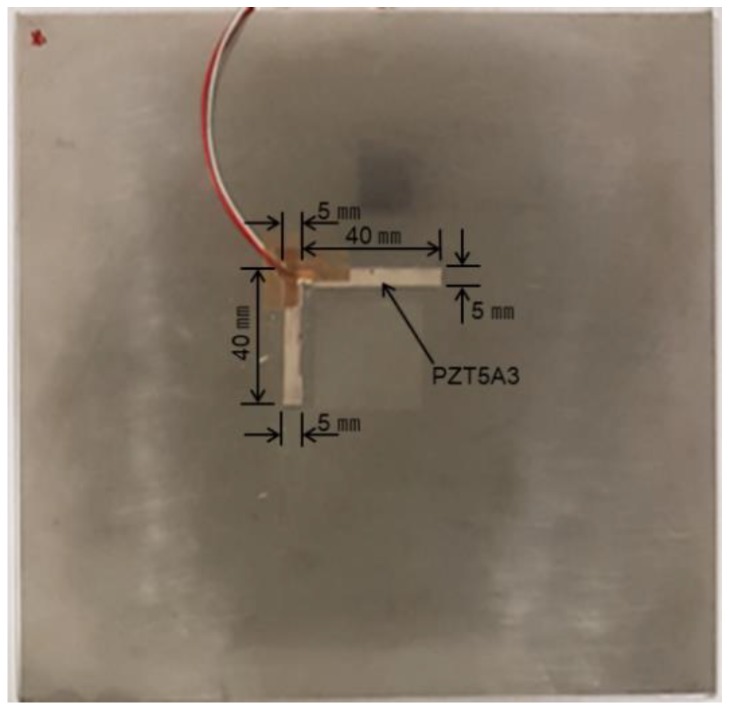
Photograph of the specimen with the L-shaped sensor.

**Figure 9:**
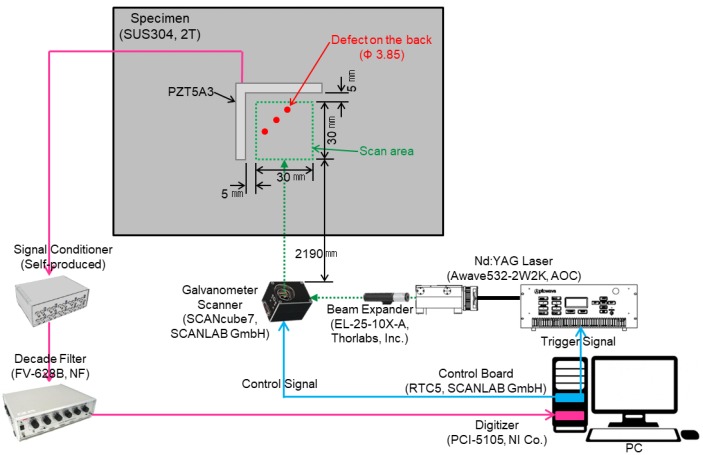
Schematic of the experimental setup using the L-shaped sensor.

**Figure 10:**
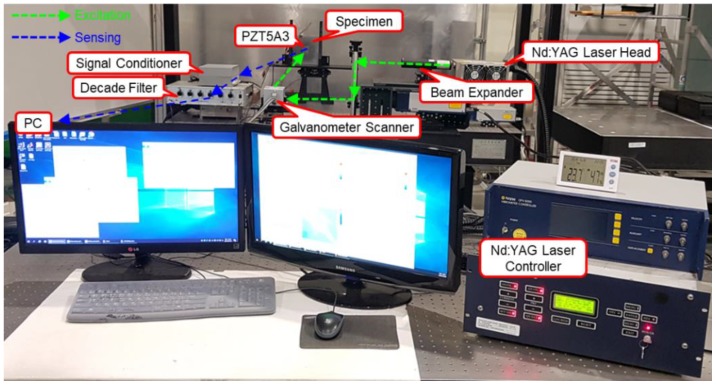
Photo of the experimental setup using the L-shaped sensor.

Three defects of ϕ3.85 sizes were made on the back of the specimen, and the L-shaped PZT5A3 sensor was attached to the specimen as shown in Figure 8. The experimental procedure is the same as that in the previous experiment. Figure 9 and Figure 10 show the schematic and photograph of the experimental setup respectively.

### 5.2. Experimental Results of Defects Visualization in the Time-Domain

**Figure 11:**
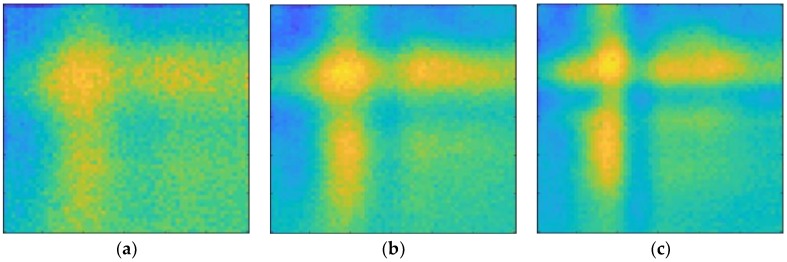
Ultrasonic propagation at different band-pass filter bandwidths: (**a**) 5 kHz–100 kHz; (**b**) 5 kHz–200 kHz; and (**c**) 5 kHz–500 kHz.

**Figure 12:**
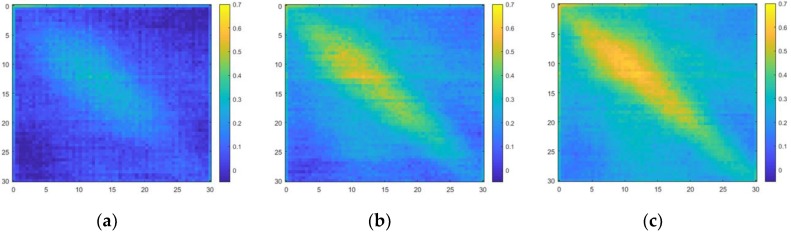
Power spectral at different band-pass filter bandwidths: (**a**) 5 kHz–100 kHz; (**b**) 5 kHz–200 kHz; and (**c**) 5 kHz–500 kHz.

The results are shown in Figure 11 and Figure 12. The result of passing through the band-pass filter with 5 kHz–50 kHz bandwidth is almost the same as the result of passing through the band-pass filter with 5 kHz–100 kHz bandwidth. The result of passing the band-pass filter with 5 kHz–50 kHz bandwidth was therefore subtracted from the figure and the result of passing the band-pass filter with 5 kHz–200 kHz bandwidth was added for a detailed comparison. It is difficult to find the defects in the ultrasonic propagation images. Moreover, the defects are also hard to distinguish from the power spectral density. Defects could not be distinguished due to the high amplitude and energy of the overlapping ultrasonic waves detected by the L-shaped sensor.

### 5.3. Algorithm for Detecting the Defects

**Figure 13:**
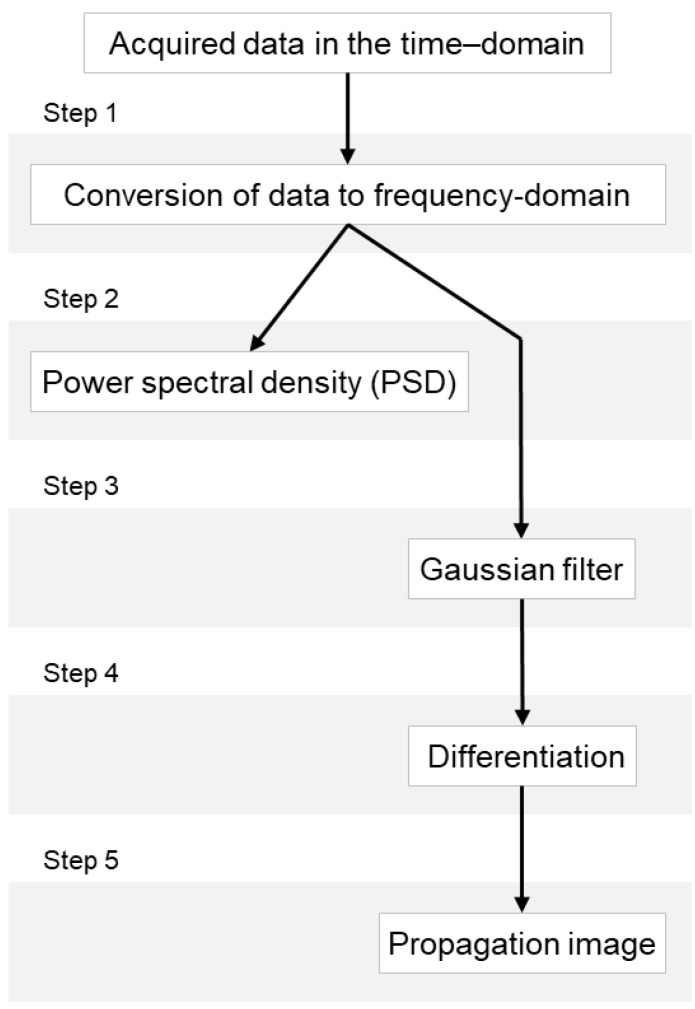
Flowchart for detecting defects.

Defect extraction is limited from the time-domain data. In order to detect defects through frequency component and phase information for each signal, the acquired data is converted from the time-domain to frequency-domain using Fourier transform. The defects are detected using two methods for the data converted to the frequency-domain. The first method is to obtain the power spectral density from the data converted to the frequency-domain using Parseval’s theorem. The defects are detected by comparing the power spectral density of the non-defective part and the defective part. The second method is to apply the Gaussian filter and differentiation to data in frequency-domain. The Gaussian filter is a linear filter that is typically used to cloud an image or reduce noise. The filter smooths the data in the form of a masking that shows the central value clearly, but not the surrounding values. The frequency-domain data after a Gaussian filter was applied was then differentiated to obtain the propagation image in order to detect defects. The flowchart of the algorithm for detecting defects is shown in Figure 13.

### 5.4. Results of Applying the Algorithm

**Figure 14:**
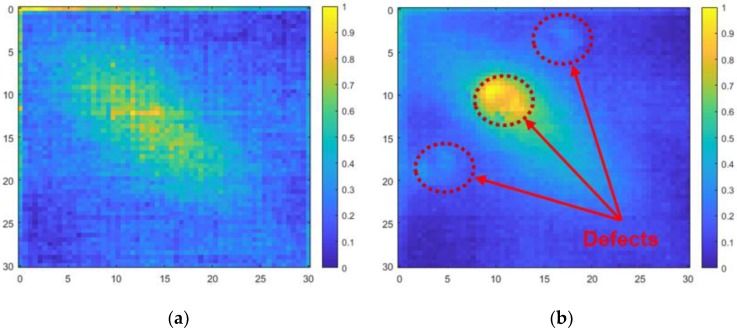
Power spectral density in the frequency-domain: (**a**) specimen without the defects; (**b**) specimen with the defects.

**Figure 15:**
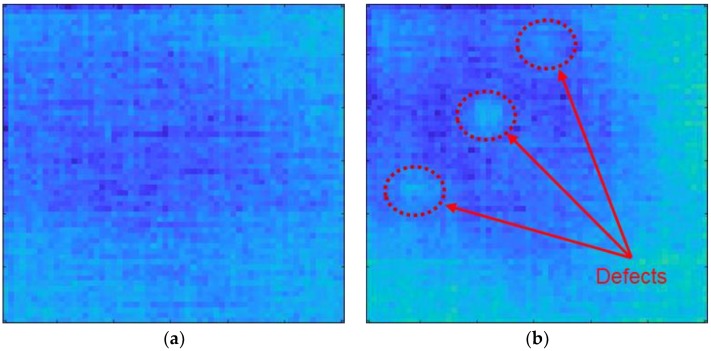
Ultrasonic propagation images in the frequency-domain: (**a**) specimen without the defects; (**b**) specimen with the defects.

The defects that did not appear in the data in time-domain could be distinguished in the frequency-domain power spectral density. Figure 14 shows a comparison of specimens with and without defects. The defects are clearly shown compared to the power spectral density in the time-domain. The defects are also easier to detect in the ultrasonic propagation images after the algorithm was applied. The ultrasonic propagation images for specimens with and without defects are shown in Figure 15.

## 6. Conclusions

In this study, defects in the specimen were detected using a Nd:YAG laser generator and a PZT5A3 line sensor and visualized using a laser ultrasonic wave generated by the thermoelastic behavior of the material. In order to overcome the disadvantages of normal single sensors and multiple sensors, an I-shaped sensor and a L-shaped sensor were used to detect defects in the steel structure. The defect detection results were visualized using the power spectral density and ultrasonic propagation images, and compared at different bandwidths of the band-pass filter.

The I-shaped sensor showed a propagation image of the axial spreading out, and the measurement area of the sensor was easily confirmed. However, the detection of the defects was made difficult by the formation of shadows behind the defects. To overcome this drawback, the L-shaped sensor was used. No shadows were cast behind the defects, but the defects could not be distinguished because of the high amplitude and power of the overlapping ultrasonic waves. To better distinguish the defects, the visualization algorithm was applied to the data acquired from the L-shaped sensor. The data in the time-domain was converted to the frequency-domain in order to distinguish the signals. The power spectral density was obtained from the frequency-domain data, and ultrasonic propagation images were obtained by applying the Gaussian filter and differentiation to the data. The defects could be more easily identified after applying the algorithm compared to when the algorithm was not applied. These results confirm that the sensors in this study detected defects more accurately and efficiently than existing sensors. In addition, defects could be more easily identified by applying the visualization algorithm.
